# Common SNPs in FTO Gene Are Associated with Obesity Related Anthropometric Traits in an Island Population from the Eastern Adriatic Coast of Croatia

**DOI:** 10.1371/journal.pone.0010375

**Published:** 2010-04-28

**Authors:** Ge Zhang, Rebekah Karns, Nina Smolej Narancic, Guangyun Sun, Hong Cheng, Sasa Missoni, Zijad Durakovic, Pavao Rudan, Ranajit Chakraborty, Ranjan Deka

**Affiliations:** 1 Center for Genome Information, Department of Environmental Health, University of Cincinnati, Cincinnati, Ohio, United States of America; 2 Department of Family and Community Medicine, University of Cincinnati, Cincinnati, Ohio, United States of America; 3 Institute for Anthropological Research, Zagreb, Croatia; 4 Center for Computational Genomics, University of North Texas Health Science Center, Fort Worth, Texas, United States of America; National Institute of Child Health and Human Development/National Institutes of Health, United States of America

## Abstract

**Background:**

Multiple studies have provided compelling evidence that the FTO gene variants are associated with obesity measures. The objective of the study was to investigate whether FTO variants are associated with a broad range of obesity related anthropometric traits in an island population.

**Methodology/Principal Findings:**

We examined genetic association between 29 FTO SNPs and a comprehensive set of anthropometric traits in 843 unrelated individuals from an island population in the eastern Adriatic coast of Croatia. The traits include 11 anthropometrics (height, weight, waist circumference, hip circumference, bicondilar upper arm width, upper arm circumference, and biceps, triceps, subscapular, suprailiac and abdominal skin-fold thicknesses) and two derived measures (BMI and WHR). Using single locus score tests, 15 common SNPs were found to be significantly associated with “body fatness” measures such as weight, BMI, hip and waist circumferences with *P*-values ranging from 0.0004 to 0.01. Similar but less significant associations were also observed between these markers and bicondilar upper arm width and upper arm circumference. Most of these significant findings could be explained by a mediating effect of “body fatness”. However, one unique association signal between upper arm width and rs16952517 (*P*-value = 0.00156) could not be explained by this mediating effect. In addition, using a principle component analysis and conditional association tests adjusted for “body fatness”, two novel association signals were identified between upper arm circumference and rs11075986 (*P*-value = 0.00211) and rs16945088 (*P*-value = 0.00203).

**Conclusions/Significance:**

The current study confirmed the association of common variants of FTO gene with “body fatness” measures in an isolated island population. We also observed evidence of pleiotropic effects of FTO gene on fat-free mass, such as frame size and muscle mass assessed by bicondilar upper arm width and upper arm circumference respectively and these pleiotropic effects might be influenced by variants that are different from the ones associated with “body fatness”.

## Introduction

The worldwide prevalence of obesity has reached epidemic proportions in recent decades and is associated with increased risks of type 2 diabetes and cardiovascular diseases. The increase in overweight and obesity has been attributed to modernization, calorie-rich nutritionally poor diets and sedentary lifestyles [1], [2]. In addition to environmental determinants, twin and adoption studies show that genetic factors strongly influence the development of obesity and its associated morbidities [3]–[6]. The reported heritability of common obesity is substantially high ranging between 30 and 70% [7]. While candidate gene studies and genome-wide linkage analysis have identified numerous obesity related loci and some of which have been replicated across different studies and populations, precise identification of obesity genes has been difficult [7]–[9]. However, recent genome-wide association studies (GWAS) have achieved remarkable success in unraveling the genetic basis of common diseases. Using this approach, common variants associated with adult and childhood obesity have been identified in several gene regions, including INSIG2, FTO, MC4R, BDNF, SH2B1 [10]–[14]. While the effect sizes of these variants contributing to the risk of obesity are modest, the fat mass obesity-associated (FTO) gene region has been replicated in several studies and in multiple populations [11], [14]–[17].

Although the aforementioned studies provide compelling evidence that the FTO gene is involved in altering fat mass, the reported associations are typically limited to “body fatness” measures such as body weight, body mass index (BMI) [11], [12], [14], [16], [18], hip and waist circumference [16], [19]. We report here the associations of common FTO variants with these classic and other obesity related anthropometric measures including bicondilar upper arm width, upper arm circumference and five skin-fold thickness measures. In all, we tested for association of 29 SNPs, eight from previous GWAS and 21 common tagging SNPs with 13 anthropometric traits in a sample of 843 unrelated adult individuals from an island population of the eastern Adriatic coast of Croatia. Our choice of the study sample for such genetic association study was prompted by their relatively homogenous genetic background and similar life style and dietary patterns. The objective of this study was to replicate the association between FTO SNPs and “body fatness” measures in our sampled population and to examine whether FTO variants affected other obesity related anthropometric measures.

## Methods

### Ethics Statement

This study was approved by the Institutional Review Board of the University of Cincinnati and the Ethics Committee of the Institute for Anthropological Research, Zagreb. Written informed consent was obtained from each study participant.

### Study population and phenotypic measurements

**Figure 1:**
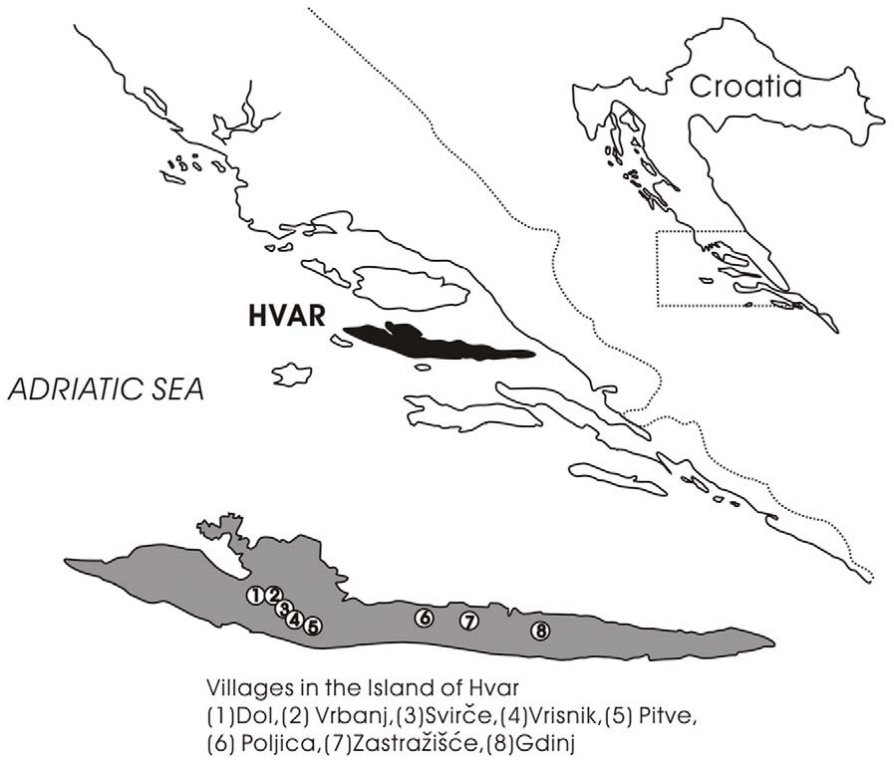
Geographical location of Hvar and the 8 villages from where the samples were collected.

The sample was derived from a study on genetics of metabolic syndrome in an isolated population from the middle Dalmatian island of Hvar in the eastern Adriatic coast of Croatia (Figure 1). The Croatian islanders are predominantly of Slavic origins who migrated from the mainland and have remained isolated since their last emigration in the 18^th^ century [20], [21]. Anthropometric data and blood samples were collected in two field seasons of May 2007 and May 2008. A total of 843 unrelated subjects (360 male and 483 female) between age 18 and 80 years residing in eight villages of the Hvar island (Figure 1) were used for this study. The anthropometric traits consist of: height (Ht), weight (Wt), waist circumference (WC), hip circumference (HC), bicondilar upper arm width (UAW), upper arm circumference (UAC), and biceps (BiS), triceps (TrS), subscapular (SbS), suprailiac (SpS) and abdominal (AbS) skin-fold thicknesses (Table 1). Anthropometric measurements were obtained by standard techniques as described in Weiner and Lourie [22].

**Table 1:** Summary statistics of anthropometric measures.

Anthropometric measures (unit)	Male (N = 360)			Female (N = 483)		
	Mean±SE	Range (min, max)	NA*	Mean±SE	Range (min, max)	NA*
Ht (cm)	177.42±0.381	(157.2, 206)	0	164.1±0.309	(139, 187.2)	0
Wt (kg)	89.31±0.682	(60.2, 142.5)	0	74.1±0.578	(43, 123.9)	0
BMI (kg/m^2^)	28.33±0.176	(19.64, 42.23)	0	27.55±0.209	(16.7, 45.79)	0
WC (cm)	101.85±0.474	(79.3, 138.5)	1	91.91±0.551	(62.3, 137.4)	1
HC (cm)	104.42±0.384	(90.8, 132)	0	106.15±0.476	(75, 150)	1
WHR	0.98±0.003	(0.727, 1.124)	1	0.87±0.003	(0.651, 1.12)	1
UAC (mm)	301.29±1.333	(235, 382)	0	289.12±1.428	(209, 400)	0
UAW (mm)	73.93±0.318	(54, 98)	0	64.55±0.282	(46, 99)	0
BiS (mm)	14.02±0.355	(1, 37.6)	1	20.43±0.342	(4.2, 39)	0
TrS (mm)	15.04±0.306	(1, 44)	1	25.95±0.294	(4.6, 43.2)	0
SbS (mm)	24.12±0.354	(8.8, 47)	3	24.39±0.371	(7.2, 49.9)	3
SpS (mm)	28.03±0.451	(9, 48.8)	2	30.26±0.388	(7.2, 52.3)	1
AbS (mm)	30.71±0.483	(7.4, 58.2)	2	33.39±0.407	(9, 59)	1

*NA: number of missing values.

### SNP selection and genotyping

The FTO gene (MIM 610966) is large encompassing >410kb of genomic region. Therefore, we adopted a targeted approach to search for common tagging SNPs within 30kb upstream and 30kb downstream of the original and most significantly associated SNP, rs9939609 reported by Frayling et al. [11]. Twenty-eight SNPs were selected by a tagging approach [23] using the Caucasian HapMap database (www.hapmap.org) based on a pairwise *r* ^2^ of ≥0.8 among all common SNPs with minor allele frequency of ≥0.05. We also included eight significant SNPs from previous GWAS (rs9939973, rs1421085, rs1121980, rs17817449, rs8050136, rs3751812, rs9939609, rs7190492). These 36 SNPs span 60kb and fall in intron 1 and intron 2 of the FTO gene. Genotyping was performed using the SNPlex protocol (Applied Biosystems), which is based on multiple oligonucleotide ligation/PCR assay with a universal ZipChute™ probe detection for high-throughput multiplexed SNP genotyping. Details of the SNPlex genotyping methods were described previously [24]. To assure genotypic quality control, negative controls and blind duplicates were introduced in each batch of samples in the 96-well format. The overall genotype call rate of the 36 SNPs was 96.5% and the genotype consistency rate based on 8 internal replicates was higher than 99.5%.

### Statistical analyses

Statistical analyses were performed using R (version 2.8.0) and the GenABEL library [25]. Pairwise LD (*r* ^2^) between markers was estimated by Haploview (version 4.1) [26]. The anthropometric measures were adjusted for age and gender and their interaction term by linear regression before conducting the cluster or association analyses. Deviation of genotype frequencies from Hardy-Weinberg equilibrium (HWE) was assessed by the exact test [27] implemented in GenABEL. The association between each SNP and quantitative anthropometric measures was evaluated by the score test [28] under the additive model. Permutation test with 100,000 replications was used to access the empirical significance adjusted for the number tests on multiple markers.

## Results

### Correlation structure and cluster of anthropometric measures

Summary statistics of the 11 anthropometrics and two derivatives (BMI and WHR) are listed in Table 1 for males (*N* = 360) and females (*N* = 483) separately. Since these measures are correlated with gender and age (data not shown), we first adjusted the effects of gender and age and their interaction term by linear regression. Unless otherwise mentioned, all data analyses were performed on these adjusted measures.

**Figure 2:**
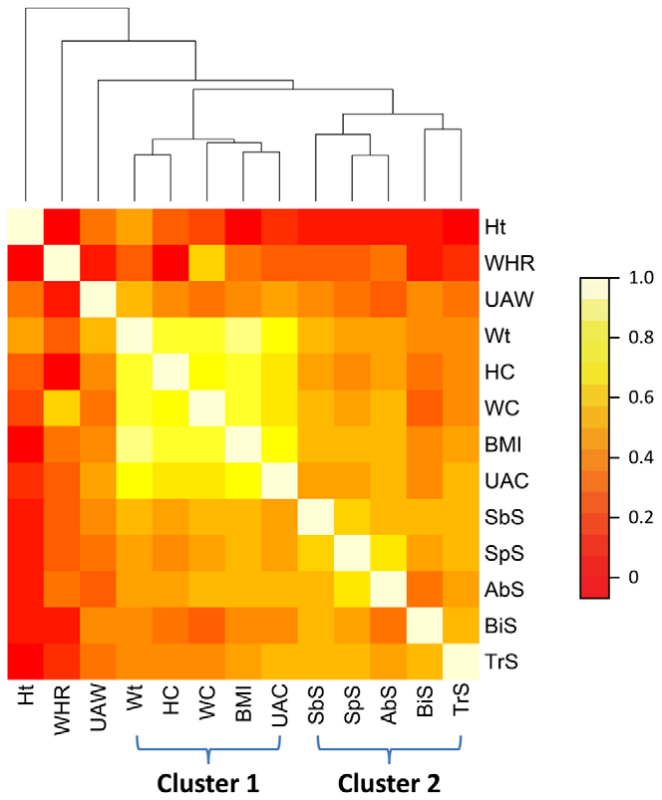
Hierarchical clustering of the 13 anthropometric traits.

The anthropometric measures we studied correlated to each other (Supplement Table S1). Therefore, we first clustered these phenotype measures based on their pairwise correlation structure using hierarchical cluster approach. As expected, the measures that reflect “body fatness” (Wt, HC, WC and BMI) together with UAC clustered together (cluster 1). Likewise, a second cluster (cluster 2) reflecting “subcutaneous obesity” was represented by the five skin fold measures. Ht, WHR and UAW were outliers to these two clusters (Figure 2).

### Genetic association analysis

**Figure 3:**
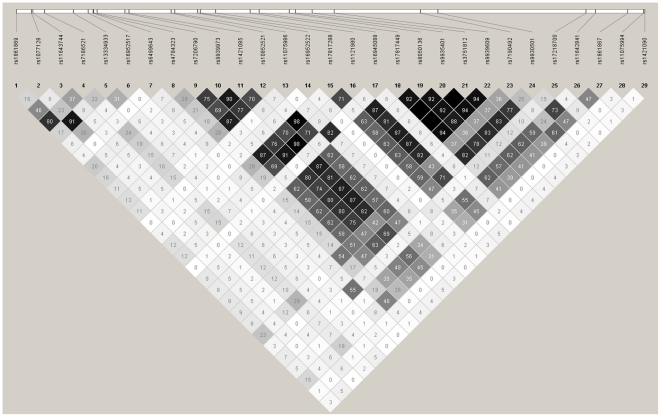
Distribution and pairwise LD (*r* ^2^) map of the 29 SNP markers.

Among the 36 genotyped SNPs, seven were excluded from the analysis either due to low minor allele frequency (5 with MAF<0.05) or deviation from HWE (2 with exact HWE test *P*-value<0.01). We tested the genetic association of the remaining 29 SNPs with each of the adjusted anthropometric trait using the single-locus score test under additive model (1df test). The distribution and the pairwise linkage disequilibrium (LD) of these 29 SNPs are shown in Figure 3. Significant associations were found between several SNPs and various phenotypic measures (Table 2). Majority of these significant associations were between the “body fatness” measures (Wt, HC, WC and BMI) of cluster 1 and a set of highly polymorphic SNP markers (MAF>0.3) that are located between rs7206790 (No. 9) and rs1861867 (No. 27), which spans approximately 50kb. For abbreviation, we refer to these 15 significant markers as “body fatness” markers (highlighted in boldface in Table 2). These associations remained significant even after multiple-testing adjustment on the number of markers by permutation test (Table 2 and Supplement Table S2).

**Table 2:** Association test between SNP markers and anthropometric traits.

No	rs id$	Pos‡	Alleles§	MAF	*P*-value of single-locus association test (1df test under additive model)*												
					Outliers			Body fatness measures and UAC (Cluster 1)					Subcutaneous obesity measures (Cluster 2)				
					Ht	WHR	UAW	Wt	HC	WC	BMI	UAC	SbS	SpS	AbS	BiS	TrS
1	rs1861869	52347682	CG	0.450	0.173	0.124	**0.00511**	**0.0182**	0.0854	**0.021**	0.0665	**0.0296**	0.147	0.143	0.179	0.998	0.235
2	rs1077128	52349154	GT	0.208	0.559	0.956	0.066	0.149	0.258	0.359	0.169	0.0792	**0.0103**	0.12	0.111	0.393	0.93
3	rs11643744	52349299	AG	0.280	0.254	0.116	0.264	0.172	0.362	0.0967	0.421	0.244	0.808	0.372	0.425	0.621	0.204
4	rs7186521	52350423	GA	0.496	0.147	0.584	**0.00644**	**0.0161**	**0.0477**	0.0555	0.0701	**0.0159**	0.11	0.0991	0.15	0.596	0.392
5	rs13334933	52353137	AG	0.195	0.311	0.799	**0.0196**	0.0628	0.135	0.292	0.11	0.108	**0.0344**	0.273	0.131	0.377	0.985
6	rs16952517	52354558	GA	0.134	0.922	0.174	**0.00156**	0.343	0.213	0.852	0.274	0.665	0.611	0.646	0.69	0.603	0.525
7	rs6499643	52355019	TC	0.152	0.12	0.879	0.991	0.435	0.373	0.656	0.926	0.833	0.446	0.943	0.71	0.399	0.657
8	rs4784323	52355066	GA	0.307	0.953	0.205	0.178	0.211	0.252	0.122	0.141	**0.00993**	0.288	0.95	0.84	0.569	0.374
9	**rs7206790**	52355409	GC	0.469	0.851	**0.00329**	**0.0181**	**0.00713**	0.0694	**0.000902**	**0.00651**	**0.00794**	**0.00702**	0.116	0.288	0.716	**0.0264**
10	**rs9939973**	52358069	GA	0.493	0.369	**0.0304**	**0.0078**	**0.00243**	**0.0139**	**0.000861**	**0.00472**	**0.00951**	**0.0404**	0.204	0.088	0.583	**0.022**
11	**rs1421085**	52358455	TC	0.465	0.609	0.127	**0.00707**	**0.0013**	**0.000997**	**0.000403**	**0.000969**	**0.0155**	**0.042**	0.359	0.246	0.635	**0.0201**
12	**rs10852521**	52362466	CT	0.450	0.815	**0.0233**	**0.0191**	**0.0203**	0.059	**0.00338**	**0.0172**	**0.00572**	**0.0125**	0.173	0.435	0.795	**0.0493**
13	rs11075986	52362845	CG	0.088	0.596	0.534	0.425	0.0622	**0.0431**	0.223	0.0684	0.632	0.736	0.991	0.245	0.645	0.416
14	rs16952522	52364999	CG	0.057	0.0641	0.307	0.192	0.255	0.166	0.0802	**0.0218**	**0.026**	**0.0212**	0.287	0.0732	0.311	**0.0124**
15	**rs17817288**	52365265	GA	0.455	0.667	**0.0119**	**0.00579**	**0.0183**	**0.0445**	**0.00165**	**0.0166**	**0.00767**	**0.00638**	0.0994	0.276	0.583	0.0614
16	**rs1121980**	52366748	CT	0.470	0.471	0.117	**0.0038**	**0.00103**	**0.00218**	**0.000621**	**0.00126**	**0.0175**	**0.0461**	0.216	0.145	0.632	**0.0157**
17	rs16945088	52370025	AG	0.075	0.472	0.233	0.175	0.131	**0.0353**	0.368	0.146	0.551	0.562	0.782	0.78	0.733	0.49
18	**rs17817449**	52370868	TG	0.437	0.258	0.146	**0.004**	**0.000906**	**0.00206**	**0.000888**	**0.00249**	0.055	0.0542	0.0937	0.0937	0.363	**0.0153**
19	**rs8050136**	52373776	CA	0.438	0.183	0.152	**0.00708**	**0.00503**	**0.00777**	**0.00262**	**0.0178**	0.0972	0.267	0.3	0.273	0.927	0.204
20	**rs9935401**	52374339	GA	0.438	0.309	0.324	**0.00395**	**0.00188**	**0.00331**	**0.00336**	**0.00475**	0.0675	0.133	0.152	0.144	0.517	**0.0227**
21	**rs3751812**	52375961	GT	0.436	0.341	0.231	**0.00879**	**0.00291**	**0.00293**	**0.00201**	**0.00637**	0.0501	0.0977	0.169	0.132	0.541	**0.0179**
22	**rs9939609**	52378028	TA	0.432	0.26	0.103	**0.00407**	**0.00181**	**0.00624**	**0.00156**	**0.0043**	0.0835	0.0757	0.102	0.0879	0.434	0.0638
23	**rs7190492**	52386253	GA	0.328	0.232	**0.0228**	**0.0144**	**0.00538**	**0.011**	**0.000892**	**0.013**	**0.00576**	0.101	0.105	0.351	0.753	0.489
24	**rs9930501**	52387953	AG	0.470	0.589	0.16	**0.00687**	**0.0128**	**0.025**	**0.00791**	**0.0171**	**0.0452**	0.329	0.2	0.148	0.693	**0.0288**
25	rs17218700	52402080	GA	0.144	0.839	0.481	0.865	0.275	0.259	0.23	0.236	0.201	0.825	0.653	0.825	0.904	0.353
26	**rs11642841**	52402988	CA	0.473	0.469	**0.0189**	0.0858	**0.0499**	0.143	**0.00938**	0.0766	0.191	0.553	0.717	0.724	0.842	0.269
27	**rs1861867**	52406062	CT	0.350	0.532	0.0556	**0.0235**	**0.00564**	**0.00664**	**0.00134**	**0.00478**	**0.0125**	0.426	0.221	0.379	0.821	0.356
28	rs11075994	52407580	GA	0.295	0.112	**0.0244**	0.789	0.382	0.265	**0.0233**	0.0844	0.0711	0.164	0.245	0.256	0.58	0.201
29	rs1421090	52407671	TC	0.228	0.346	0.164	0.502	0.67	0.941	0.329	0.892	0.572	0.197	0.475	0.125	0.867	0.831

*Nominal significant *P* values (<0.05) were shown in bold font. *P* values that can pass permutation test were underlined.

$Markers significantly associated with multiple body fatness measures (“body fatness” markers) were bolded.

‡Chromosome positions of the SNPs are based on Human Reference Genome Sequence Build 36.

§The alleles were shown as major allele/minor allele.

Interestingly, UAW was found to be significantly associated with “body fatness” markers, although it is less closely correlated with the “body fatness” traits (Figure 2). In addition, one significant association signal was found between UAW and rs16952517 (No. 6, *P*-value = 0.00156, significant even with permutation test). In contrast, “body fatness” markers were less significantly associated with UAC (a member of cluster 1 traits) compared with UAW. WHR and two skin fold measures (TrS and SbS) of cluster 2 also showed nominally significant associations with some of these markers, significance of which were not supported by the permutation test.

Since multiple cluster 1 measures and UAW were found to be associated with similar sets of SNP markers, we performed a principle component analysis (PCA) to evaluate whether some “lower-dimensional summary” of these variables provide more information about the observed associations. We identified three major components (PC1, PC2 and PC3) that could explain up to 90% of the total variance of these measures (Supplement Table S3).

**Figure 4:**
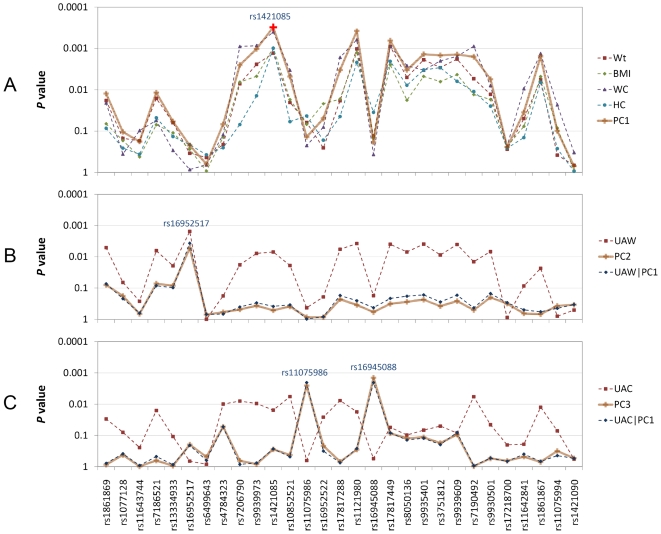
*P* values of association tests. A) Association test *P* values between “body fatness” measures (Wt, BMI, WC, HC) and PC1; B) Association test *P* values of UAW, PC2 and PC1 adjusted UAW (UAW|PC1); C) Association test *P* values of UAC, PC3 and PC1 adjusted UAC (UAC|PC1).

The first component (PC1) explained more than 70% of the total variance and was approximately an average of all the included measures with similar loading coefficients (around −0.40∼−0.45), except for UAW (with a loading coefficient of −0.264). The association signals (*P*-values) observed between PC1 and the “body fatness” markers shared similar pattern with individual body fatness measures (Wt, HC, WC and BMI) and the highest association peak was found at rs1421085 (*P*-value = 0.000307) (Figure 4A). Therefore, we considered PC1 as a summary variable for “body fatness” and it well captured the significant association between individual “body fatness” measures and “body fatness” markers. To confirm this, we tested association using PC1 as covariate to remove the effect of “body fatness”. As expected, all the significant signals found between “body fatness” measures and “body fatness” markers disappeared (*P*-value>0.01) after the adjustment for PC1 (Supplement Figure S1). We also performed association test conditional on rs1421085 (the most significant marker) to investigate whether the significant association signals between “body fatness” measures and “body fatness” markers can be explained by a single locus. Again, after adjustment for rs1421085, the significant signals on other “body fatness” markers diminished (Supplement Figure S2).

The second component (PC2) had a prominent UAW constituent (with a loading coefficient of 0.935) and it mainly represented the effect of UAW with adjustments of other variables. Not surprisingly, PC2 was not associated with “body fatness” markers because PC2 was independent to PC1 and the later already absorbed most of the variance of “body fatness”. In this sense, PC2 could be approximately regarded as UAW adjusted for PC1 or “body fatness” (UAW|PC1 in Figure 4B), which was exemplified by the similar association pattern of these two variables. Interestingly, unlike the “body fatness” markers, the unique significant association between rs16952517 and UAW still hold for PC2 (*P*-value = 0.00531) and PC1 adjusted-UAW (*P*-value = 0.00372).

Similar to PC2, the third component (PC3) mainly represents the effect of UAC (with a loading coefficient of −0.878) and could be approximately regarded as UAC adjusted for PC1 (or “body fatness”) represented as UAC|PC1 in Figure 4C. Accordingly, PC3 was not associated with the “body fatness” markers. However, two novel significant association signals emerged between PC3 and rs11075986 (*P*-value = 0.00255) and rs16945088 (*P*-value = 0.00142). Similar association signals exist between PC1 adjusted UAC (UAC|PC1) and rs11075986 (*P*-value = 0.00211) and rs16945088 (*P*-value = 0.00203).

## Discussion

We investigated genetic association between a set of obesity related anthropometric measures and 29 common SNPs in the FTO gene. These markers were selected either based on significant findings of previous GWAS or by pairwise tagging approach. The anthropometric measures we studied were grouped into two major clusters: the first cluster included four closely correlated “body fatness” measures [29] and UAC which is usually used as an anthropometric measurement of muscle mass [30] or nutrition status [31]; the second cluster included five skin fold measures for “subcutaneous obesity”. Ht, WHR and UAW were loosely correlated with these two clusters.

Although the sample size of present study was relatively small compared with the previous GWAS [11], [12], [14], [16], [18], [32], the number of association tests in our study was restricted to a limited number of SNP markers in the FTO gene. In addition, the current study benefited from a homogenous genetic background and environmental exposure (similar life style and dietary pattern) of the Croatian islanders [20], [21], [33]. We have not detected any signature of population substructure by the Structure program [34], [35] based on multi-locus genotype data of ∼60 SNPs on different chromosomes (data not shown). Our study was therefore sufficiently powered to validate the association between FTO markers and obesity related anthropometric measures. Our power analysis (one-way ANOVA power analysis for quantitative trait with 1df for allelic test) indicated that our study should have 94% and 74% power to detect SNPs with a locus-specific heritability of 1.5% at the nominal significance level (α = 0.05) or the significance level adjusted for the number of independent tests of multiple SNPs (α = 0.05/16), respectively. The 29 SNPs are in close LD and the estimated number of equivalent independent tests is around 16 by permutation analysis.

Consequently, we confirmed association between a set of high frequency (MAF>0.3) SNPs in FTO gene and “body fatness” measures in our samples. Specifically, the most significant associations were observed with six previously reported GWAS SNPs including rs1421085 [36], rs1121980 [12], [37], rs17817449 [38], rs8050136 [14], [32], [39]–[41], rs3751812 [17] and rs9939609 [11], [18], [42], [43] as well as with several newly identified high frequency tagging SNPs (rs7206790, rs9939973, rs10852521, rs17817288, rs9935401, rs7190492, rs9930501, rs11642841 and rs1861867). Among those, rs9930501 can surrogate two other SNPs reported by previous GWAS: rs9930506 [16] and rs9941349 [44] with perfect (*r* ^2^ = 1.0) or near perfect (*r* ^2^ = 0.961) LD based on the HapMap data of Caucasian samples (CEU). Since these significant SNPs are in strong pairwise LD (average *r* ^2^ = 0.65) and show similar association patterns with different “body fatness” measures, we referred these SNPs as “body fatness” markers. Conditional association analysis on rs1421085 indicated no major effect of allelic heterogeneity on “body fatness” measures, since adjusting for rs1421085 completely eliminated the associations between “body fatness” measures and “body fatness” markers.

The estimated effect size of these “body fatness” markers on “body fatness” measures from our sample were comparable but of higher magnitude compared to those of previous reports [11], [14], [16], [18]. Using rs1421085 as an example, each copy of C allele was associated with an increase of age and gender adjusted Wt = 1.98 kg (15.7%SD), HC = 1.42 cm (16.0%SD), WC = 1.78 cm (17.3%SD) and BMI = 0.637 kg/m^2^ (16.1%SD) which correspond to the explained variances (under additive model) of 1.23% (Wt), 1.27% (HC), 1.49% (WC) and 1.29% (BMI) respectively. The higher effect size estimates we obtained from the present study may probably be attributed to the relatively homogenous genetic background and environmental exposures in our study population.

Interestingly, similar significant association was observed between UAW and the “body fatness” markers, although UAW is loosely correlated with “body fatness” measures. UAW, also known as elbow breadth, is a robust measure for frame size and has been suggested to be used for the interpretation of body weight [45], [46] and shown to be positively associated with total body fat as well as fat-free mass [47]. Therefore, this association might indicate a general effect of “body fatness” markers of FTO gene on body growth – not only fat mass accumulation but also the fat-free mass. This observation was consistent with several previously reported associations between lean body mass and FTO [11], [48], [49]. In addition, we also observed one additional SNP, rs16952517, which was exclusively associated with UAW but not with other anthropometric measures. This unique association might reflect possible pleiotropic effect of the FTO gene on frame size independent of the “body fatness” markers.

UAC, WHR and two skin fold measures (TrS and SbS) only showed nominal significant associations with some of these “body fatness” markers. The effect of these “body fatness” SNPs on UAC, WHR and two skin fold measures (TrS and SbS) appeared to be mediated through adiposity, since adjusting for PC1 (as a surrogate measure for “body fatness”) completely eliminated the associations (data not shown).

Through principle component analysis of the four “body fatness” measures together with UAC and UAW, we identified three major components. The first component (PC1) could be regarded as a summary for “body fatness” measures and it could surrogate the associations between “body fatness” measures and “body fatness” markers, since adjusting for this component completely eliminated the associations between “body fatness” traits and “body fatness” markers. The second and third components mainly represented the PC1 or “body fatness” adjusted UAW and UAC, respectively. Of particular note were the association between PC2 (or PC1 adjusted UAW) and rs16952517 and associations between PC3 (or PC1 adjusted UAC) and rs11075986 and rs16945088. These significant associations demonstrated that variants other than the “body fatness” markers of FTO gene might have pleiotropic effects on frame size (UAW) and muscle mass (UAC) and these effects are independent from the effect of the FTO gene on “body fatness”, which are influenced by a different set of markers.

In summary, our study confirmed the association of common variants of the FTO gene with body fatness measures in an island population from eastern Adriatic coast of Croatia. In addition to the previously reported SNPs, we identified a set of high frequency tagging SNPs associated with body fatness measures with similar magnitudes of significance. The associations between “body fatness” traits and the “body fatness” markers could be explained by a single SNP without any clear sign for allelic heterogeneity. Based on principle component analysis and conditional association tests adjusted for “body fatness” measures, we observed evidences to support the pleiotropic effects of FTO gene on fat-free mass, such as frame size and muscle mass as indicated by UAW and UAC, respectively. These possible pleiotropic effects might be influenced by variant(s) different from the ones associated with “body fatness”. This new observation is at least partially consistent with results from recent functional studies of the FTO gene in mice [50], [51], in which significant differences were observed between the *Fto* null mice [50] and *Fto^I367F^* mice [51], which suggested pleiotropic effects due to different mutations. In a recent review article [52], the possible pleiotropic effect of FTO gene on multiple quantitative traits has been illustrated (Box 2 of [52]) to support the notion that genetic variants that are implicated in complex traits are associated with multiple quantitative traits at every level of analysis.

It should be noted that although our genetic association tests and principle component analysis provide strong evidence to support pleiotropic effects of the FTO gene on fat-free mass, these results should be interpreted with caution. Considering the possibilities of statistical fluctuation due to inadequate power and multiple comparisons, further replication studies in independent populations and functional studies are required to confirm these observations. In addition, the 29 common SNPs we examined encompass only a small fraction of the FTO gene (intron 1 and intron 2). Therefore, we cannot rule out the possibility that the true causal or functional SNP(s) might be located in other regions of the gene that are in high LD with the SNPs in our study. These limitations call for additional validation in an independent sample with detailed anthropometric measurements and more comprehensive studies to clarify the pleiotropic effects of the FTO gene. These should include deep sequencing to identify the specific causal variant(s) and functional studies to further examine the multifaceted mechanism by which FTO influences body growth and energy homeostasis.

## Supporting Information

Table S1 Pairwise correlation of anthropometric measures.
(0.06 MB PDF)

Table S2 Permutation test results between SNP markers and anthropometric traits.
(0.16 MB PDF)

Table S3 Principle components of “body fatness” phenotypic measures.
(0.08 MB PDF)

Figure S1 *P* values of association tests between “body fatness” measures (Wt, BMI, WC, HC) adjusted by PC1.
(0.50 MB TIF)

Figure S2 *P* values of association tests between “body fatness” measures (Wt, BMI, WC, HC) and PC1 adjusted for rs1421085.
(0.52 MB TIF)
