# Supplementary material for: Common SNPs in FTO Gene Are Associated with Obesity Related Anthropometric Traits in an Island Population from the Eastern Adriatic Coast of Croatia
Source: PLoS One. 2010 Apr 28;5(4):e10375. doi: 10.1371/journal.pone.0010375 (PMC2860984; doi:10.1371/journal.pone.0010375)
Supplement: Table S1 — Pairwise correlation of anthropometric measures. (0.06 MB PDF) [file pone.0010375.s001.pdf]

Table S1. Pairwise correlation of anthropometric measures

	Ht	Wt	BMI	WC	HC	WHR	UAC	UAW	BiS	TrS	SbS	SpS	AbS
Ht	1.000	0.605	-0.025	0.297	0.026	0.375	0.206	0.545	-0.264	-0.468	-0.014	-0.030	-0.086
Wt	0.605	1.000	0.776	0.823	0.647	0.503	0.721	0.648	0.085	-0.057	0.441	0.360	0.362
BMI	-0.025	0.776	1.000	0.804	0.807	0.328	0.746	0.387	0.317	0.299	0.558	0.478	0.522
WC	0.297	0.823	0.804	1.000	0.676	0.721	0.678	0.509	0.034	-0.018	0.451	0.354	0.438
HC	0.026	0.647	0.807	0.676	1.000	-0.019	0.623	0.283	0.307	0.347	0.453	0.404	0.482
WHR	0.375	0.503	0.328	0.721	-0.019	1.000	0.340	0.430	-0.242	-0.350	0.188	0.101	0.142
UAC	0.206	0.721	0.746	0.678	0.623	0.340	1.000	0.471	0.260	0.286	0.491	0.441	0.506
UAW	0.545	0.648	0.387	0.509	0.283	0.430	0.471	1.000	0.045	-0.202	0.293	0.170	0.113
BiS	-0.264	0.085	0.317	0.034	0.307	-0.242	0.260	0.045	1.000	0.652	0.531	0.456	0.382
TrS	-0.468	-0.057	0.299	-0.018	0.347	-0.350	0.286	-0.202	0.652	1.000	0.417	0.471	0.457
SbS	-0.014	0.441	0.558	0.451	0.453	0.188	0.491	0.293	0.531	0.417	1.000	0.606	0.533
SpS	-0.030	0.360	0.478	0.354	0.404	0.101	0.441	0.170	0.456	0.471	0.606	1.000	0.716
AbS	-0.086	0.362	0.522	0.438	0.482	0.142	0.506	0.113	0.382	0.457	0.533	0.716	1.000
