# Supplementary material for: Common SNPs in FTO Gene Are Associated with Obesity Related Anthropometric Traits in an Island Population from the Eastern Adriatic Coast of Croatia
Source: PLoS One. 2010 Apr 28;5(4):e10375. doi: 10.1371/journal.pone.0010375 (PMC2860984; doi:10.1371/journal.pone.0010375)
Supplement: Table S2 — Permutation test results between SNP markers and anthropometric traits. (0.16 MB PDF) [file pone.0010375.s002.pdf]

Table S2. Permutation test results between SNP markers and anthropometric traits

No	rs id	Pos‡	Alleles§	MAF	Permutation *P*-value of single-locus association test (1df test under additive model)*												
					Outliers			Body fatness measures and UAC (Cluster 1)					Subcutaneous obesity measures (Cluster 2)				
					Ht	WHR	UAW	Wt	HC	WC	BMI	UAC	SbS	SpS	AbS	BiS	TrS
1	rs1861869	52347682	CG	0.450	0.904	0.813	0.0792	0.236	0.685	0.262	0.594	0.344	0.862	0.856	0.912	1	0.962
2	rs1077128	52349154	GT	0.208	1	1	0.595	0.867	0.974	0.996	0.9	0.658	0.147	0.804	0.775	0.998	1
3	rs11643744	52349299	AG	0.280	0.973	0.79	0.977	0.904	0.996	0.728	0.999	0.968	1	0.997	0.999	1	0.939
4	rs7186521	52350423	GA	0.496	0.862	1	0.0971	0.213	0.484	0.532	0.613	0.211	0.771	0.74	0.869	1	0.998
5	rs13334933	52353137	AG	0.195	0.989	1	0.251	0.577	0.837	0.986	0.773	0.766	0.387	0.98	0.829	0.997	1
6	rs16952517	52354558	GA	0.134	1	0.906	**0.027**	0.995	0.947	1	0.981	1	1	1	1	1	1
7	rs6499643	52355019	TC	0.152	0.802	1	1	0.999	0.997	1	1	1	0.999	1	1	0.998	1
8	rs4784323	52355066	GA	0.307	1	0.94	0.912	0.946	0.971	0.807	0.851	0.142	0.985	1	1	1	0.997
9	rs7206790	52355409	GC	0.469	1	0.0536	0.235	0.107	0.612	**0.0154**	0.0981	0.117	0.105	0.794	0.985	1	0.317
10	rs9939973	52358069	GA	0.493	0.996	0.355	0.115	**0.04**	0.189	**0.0149**	0.0745	0.137	0.435	0.942	0.694	1	0.275
11	rs1421085	52358455	TC	0.465	1	0.82	0.105	**0.0224**	**0.0172**	**0.00728**	**0.0177**	0.206	0.446	0.996	0.969	1	0.257
12	rs10852521	52362466	CT	0.450	1	0.289	0.246	0.257	0.556	0.0537	0.224	0.087	0.173	0.907	0.999	1	0.494
13	rs11075986	52362845	CG	0.088	1	1	0.999	0.574	0.453	0.956	0.605	1	1	1	0.969	1	0.999
14	rs16952522	52364999	CG	0.057	0.585	0.989	0.928	0.973	0.896	0.661	0.271	0.313	0.268	0.984	0.629	0.99	0.171
15	rs17817288	52365265	GA	0.455	1	0.168	0.088	0.237	0.462	**0.0274**	0.217	0.113	0.0968	0.741	0.981	1	0.568
16	rs1121980	52366748	CT	0.470	1	0.793	0.0606	**0.0179**	**0.0361**	**0.011**	**0.0223**	0.227	0.475	0.952	0.86	1	0.209
17	rs16945088	52370025	AG	0.075	1	0.962	0.909	0.828	0.393	0.997	0.86	1	1	1	1	1	1
18	rs17817449	52370868	TG	0.437	0.974	0.861	0.0635	**0.0159**	**0.0344**	**0.0152**	**0.0413**	0.532	0.528	0.72	0.717	0.997	0.205
19	rs8050136	52373776	CA	0.438	0.917	0.872	0.105	0.0793	0.115	**0.0424**	0.23	0.73	0.978	0.988	0.98	1	0.939
20	rs9935401	52374339	GA	0.438	0.989	0.992	0.0629	**0.0314**	0.0531	0.0535	0.0748	0.602	0.832	0.874	0.858	1	0.282
21	rs3751812	52375961	GT	0.436	0.994	0.96	0.127	**0.0475**	**0.0477**	**0.0332**	0.0965	0.501	0.731	0.901	0.832	1	0.234
22	rs9939609	52378028	TA	0.432	0.975	0.75	0.0644	**0.0305**	0.0947	**0.0261**	0.0683	0.677	0.642	0.749	0.694	0.999	0.581
23	rs7190492	52386253	GA	0.328	0.962	0.284	0.196	0.0842	0.156	**0.0153**	0.178	0.0874	0.741	0.762	0.995	1	1
24	rs9930501	52387953	AG	0.470	1	0.885	0.103	0.176	0.305	0.115	0.223	0.467	0.993	0.938	0.865	1	0.339
25	rs17218700	52402080	GA	0.144	1	1	1	0.981	0.974	0.961	0.963	0.936	1	1	1	1	0.995
26	rs11642841	52402988	CA	0.473	1	0.245	0.689	0.498	0.854	0.134	0.645	0.925	1	1	1	1	0.978
27	rs1861867	52406062	CT	0.350	1	0.536	0.29	0.0878	0.1	**0.0226**	0.0752	0.173	0.999	0.956	0.997	1	0.996
28	rs11075994	52407580	GA	0.295	0.78	0.299	1	0.998	0.976	0.285	0.679	0.62	0.892	0.969	0.974	1	0.936
29	rs1421090	52407671	TC	0.228	0.994	0.892	1	1	1	0.993	1	1	0.934	1	0.815	1	1

* Significant empirical *P* values (<0.05) were shown in bold font.

‡ Chromosome positions of the SNPs are based on Human Reference Genome Sequence Build 36.

§ The alleles are shown as major allele/minor allele
