# Supplementary material for: Common SNPs in FTO Gene Are Associated with Obesity Related Anthropometric Traits in an Island Population from the Eastern Adriatic Coast of Croatia
Source: PLoS One. 2010 Apr 28;5(4):e10375. doi: 10.1371/journal.pone.0010375 (PMC2860984; doi:10.1371/journal.pone.0010375)
Supplement: Table S3 — Principle components of “body fatness” phenotypic measures. (0.08 MB PDF) [file pone.0010375.s003.pdf]

Table S3. Principle components of “body fatness” phenotypic measures

Component	Variance %	Variable loadings					
		UAW	Wt	HC	WC	BMI	UAC
PC1	72.96%	-0.264	-0.452	-0.427	-0.425	-0.447	-0.403
PC2	13.01%	**0.935**	-0.011	-0.140	-0.276	-0.171	0.028
PC3	5.98%	0.131	0.204	0.382	0.154	-0.003	**-0.878**
