# Supplementary figures and images for: Common SNPs in FTO Gene Are Associated with Obesity Related Anthropometric Traits in an Island Population from the Eastern Adriatic Coast of Croatia
Source: PLoS One. 2010 Apr 28;5(4):e10375. doi: 10.1371/journal.pone.0010375 (PMC2860984; doi:10.1371/journal.pone.0010375)

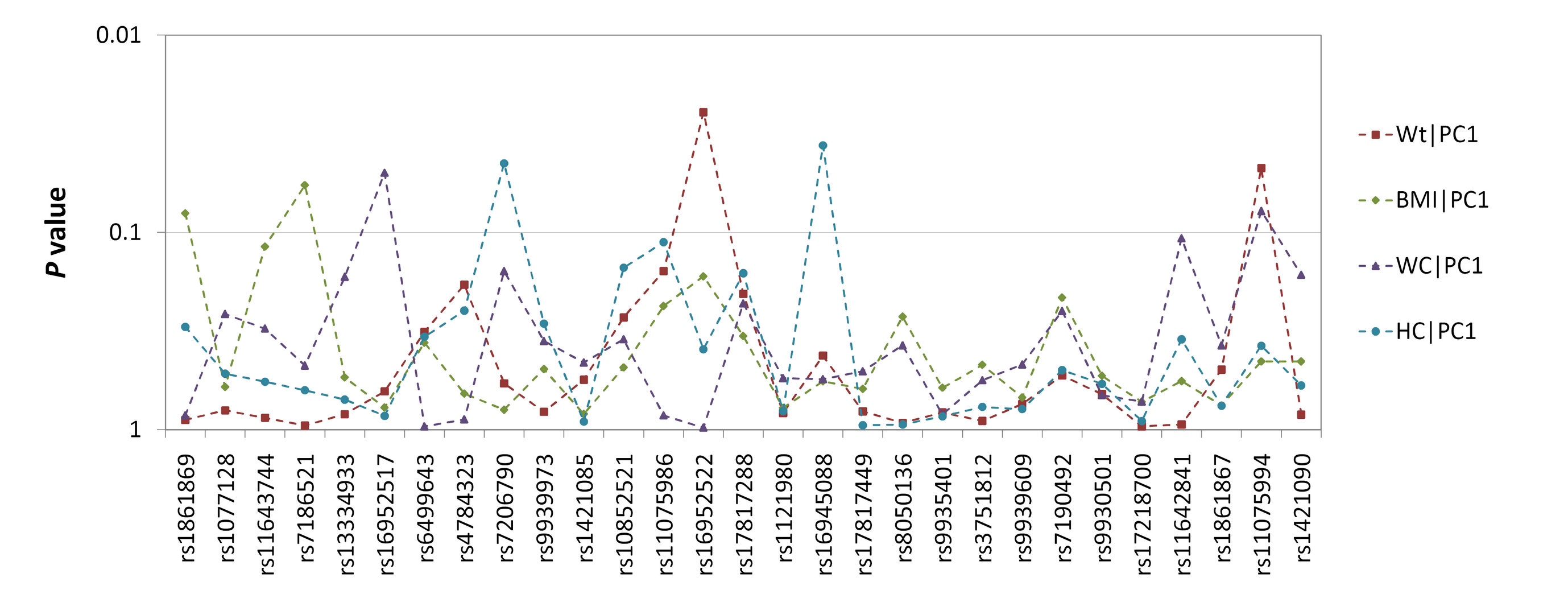

Supplement: Figure S1 — P values of association tests between “body fatness” measures (Wt, BMI, WC, HC) adjusted by PC1. (0.50 MB TIF) [file pone.0010375.s004.tif]

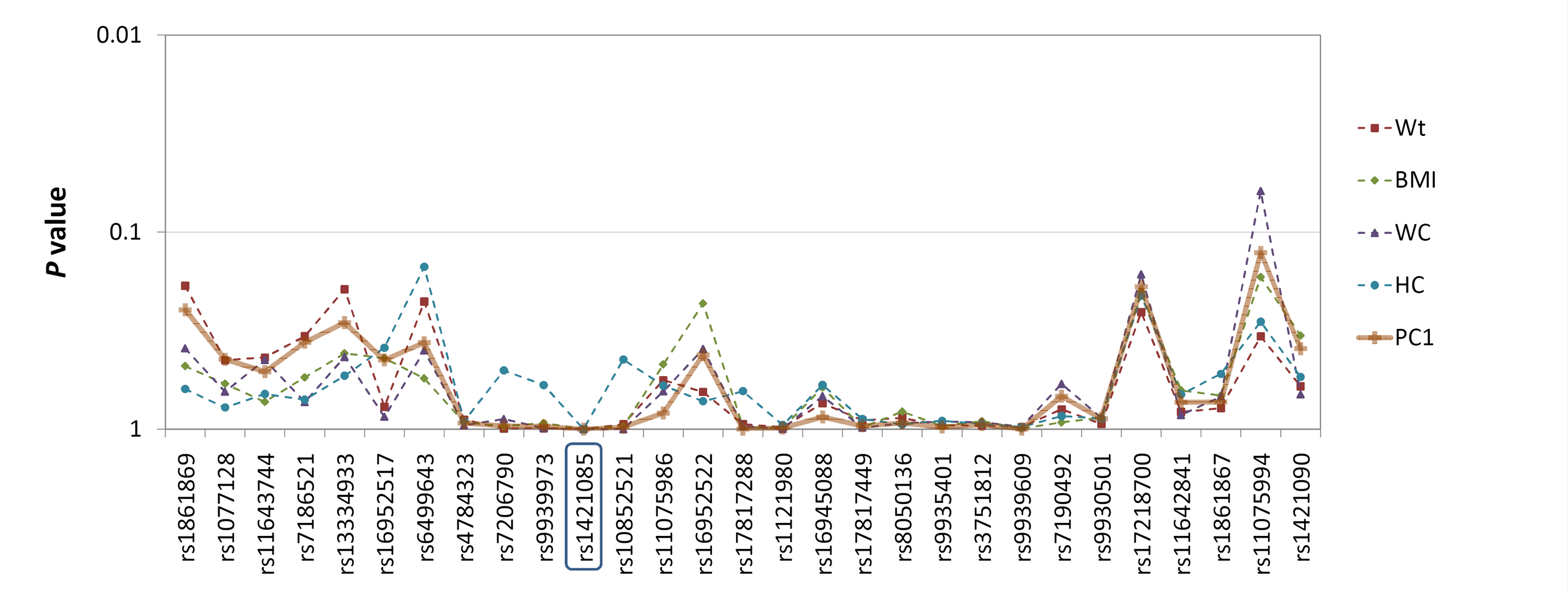

Supplement: Figure S2 — P values of association tests between “body fatness” measures (Wt, BMI, WC, HC) and PC1 adjusted for rs1421085. (0.52 MB TIF) [file pone.0010375.s005.tif]
